# Postgraduate education in healthy and active ageing: learning needs, curriculum and expected outcomes: a scoping review protocol

**DOI:** 10.12688/hrbopenres.13444.2

**Published:** 2022-10-12

**Authors:** Daisy Wiggin, Benjamin Penič, Outi Sulopuisto, Annalisa Setti, Jana Mali, Andrea Stitzel, Raija Kuisma, Fátima Baptista, Tuula Kukkonen, Olympia Konstantakopolou, Liisa Timonen, Filomena Carnide, Venetia-Sofia Velanoki, Daniela Elisabeth Ströckl, Vera Zymbal, Graça Cardadeiro, Elina Nevala, Daphne Kaitelidou, Panayota Sourtzi, Valentina Hlebec, Maša Filipovič Hrast, Suzanne Timmons

**Affiliations:** 1 Centre for Gerontology and Rehabilitation, School of Medicine, University College Cork, Cork, Ireland; 2 Faculty of Social Work, University of Ljubljana, Ljubljana, Slovenia; 3 Department of Health Sciences and Social Work, Carinthia University of Applied Sciences, Klagenfurt, Austria; 4 School of Applied Psychology, University College Cork, Cork, Ireland; 5 Faculty of Health Care and Social Services, Carinthia University of Applied Sciences, Villach, Austria; 6 Karelia University of Applied Sciences, Tikkarine 9, Joensuu, Finland; 7 CIPER, Faculdade de Motricidade Humana, Universidade de Lisboa, Lisbon, Portugal; 8 Department of Nursing, Laboratory of Health Services Organisation & Evaluation, National and Kapodistrian University of Athens, Athens, Greece; 9 Department of Nursing, Sector of Public Health, National and Kapodistrian University of Athens, Athens, Greece; 10 IARA – Institute for Applied Research on Ageing, Dep. Health and Assistive Technologies, School of Medical Engineering, Carinthia University of Applied Sciences, Klagenfurt, Austria; 11 Department of Nursing, Laboratory of Prevention, National and Kapodistrian University of Athens, Athens, Greece; 12 Faculty of Social Sciences, University of Ljubljana, Ljubljana, Slovenia

**Keywords:** learning needs, needs analysis, healthy and active ageing, age-friendly society, education

## Abstract

**Background:** As the European population ages, it becomes increasingly important to promote and facilitate healthy and active ageing and age-friendly societies. Professionals across a range of disciplines and sectors need knowledge and skills to support both.

**Objective:** This scoping review aims to identify and map the literature on learning needs, learning outcomes and respective curricula in healthy and active ageing and age-friendly society concepts.

**Inclusion criteria:** Studies focused on the teaching/learning process in healthy and active ageing and/or age-friendly society, of any design type, are eligible. Included studies may focus on undergraduate, postgraduate or continuing education and on any aspect of the educational process, such as needs analysis, content delivery, learner satisfaction/acceptability, or education outcome.

**Methods:** This review will follow the Joanna Briggs Institute (JBI) methodology for conducting scoping reviews. Four electronic databases, PubMed, EBSCO (Academic Search Complete), Scopus and Applied Social Sciences Index and Abstracts (ASSIA), will be searched, limited to studies published from 1 ^st^ January 2000. Text language will be limited to English, German, Greek, Portuguese, Finnish, and Slovenian. Google Scholar and Research Gate will be searched for grey literature, limited to the first 50 results of each. Title and abstract screening, followed by full-text screening will be undertaken independently by at least two reviewers. The JBI extraction tool will be adapted for data extraction. Quality assessment will be conducted using a tool developed by Hawker and colleagues. A narrative synthesis will outline the data in relation to the aims and objectives outlined.

## Introduction

Our society is experiencing an increase in older populations ^1^. Those aged 65 and over currently make up 20.3% of the European population, which is expected to increase to almost one third by 2100 ^2^. Increasing longevity is very welcome, but the challenges of an ageing demographic are well documented, such as a potential increased demand on health and social care systems ^3^, reduced investment capital, rising demands on pension schemes, and decreases in labour supply ^4^. While longevity can be associated with increasing quality of life, this is dependent on satisfactory physical, cognitive and mental health, and good relationships and social participation ^5^. These latter factors may be a challenge in communities low in social capital, where typical family structures mean lower intergenerational support today than in the past ^6^.

Healthy and active ageing (HAA) and age-friendly society concepts provide a multidimensional framework to not just address the challenges of an ageing demographic, but to profit from the success of greater longevity and value the potential ongoing contribution of older people to society. HAA encompasses healthy lifestyle promotion, comprising of nutritional, physical activity and social practices throughout the life course ^7^. An age-friendly society fosters HAA by building and maintaining intrinsic capacity across the life course and enabling greater functional ability in a person of any level of capacity ^1^. Combined, HAA and an age-friendly society involve many sectors, including but not limited to: health, transport, housing, labour, social protection, information and communication. They also require the action of many: government officials, service providers, industry professionals, civil society, older people and their representative organisations, families and friends.

A competent workforce in all sectors is needed to address the challenges and capitalise on an ageing Europe in a positive, comprehensive and meaningful way ^8^. Crucial to educating the future workforce is assessing learning needs, to inform the development of relevant and applicable competencies and learning outcomes for educational curricula ^9^. This scoping review aims to gain an understanding of the learning needs in HAA and age-friendly society.

Scoping reviews are an effective method to establish the extent of the research available on a particular topic, and thus identify knowledge and evidence gaps ^10^. Therefore a scoping review will be conducted with the following objectives:

1. To identify literature concerning learning needs, learning outcomes, educational content, evaluation, or processes, with respect to healthy and active ageing and age-friendly society concepts.
2. To assess the quality of the included studies and, if indicated, generate improved evidence recommendations.
3. To ultimately inform the development of a new postgraduate degree in Active Ageing and Age Friendly Society (Project website: https://www.emma-master.eu/).

## Inclusion criteria

In line with the Joanna Briggs Institute (JBI) guidelines, the initial criteria will be broad, with the intention of creating inclusion and exclusion criteria during the search process, if appropriate. To capture as much relevant data as possible, the inclusion criteria will be limited to any study which reports on student learning needs (e.g. undergraduate, postgraduate, continuing professional development) in relation to HAA and age-friendly society. In the event where a publication is deemed relevant from abstract screening, but access to the full-text is not available, authors will be contacted to request the full-text article. An overview of this criteria is found in Table 1.

**Table 1.** Inclusion and exclusion criteria.

Inclusion criteria	Exclusion criteria
• Any study design or education provider type • Populations or samples must be professionals undertaking continuing education, or undergraduate or postgraduate students • Must report on learning needs, or learning/ assessment processes, or delivery, or relevance, or acceptability, or outcome • Must be relevant to healthy and/or active ageing as a concept • Published after 1 ^st^ January 2000	• Study does not report on learning needs in the field of active ageing (e.g. study reports on healthy and/or active ageing, but not learning needs; or study reports on learning needs but not related to healthy and/or active ageing) • Full-text publications in a language other than: English, Slovenian, Portuguese, Finnish, German, or Greek • Full-text publication is not available after contacting authors • Study is focused on second-level or high school education • Study is reporting on public audiences (i.e. raising public awareness about healthy and/or active ageing) • Study is primarily focused on life-long learning/older people as learners

### Population

Postgraduate students are of primary interest in this review; it is expected that they have a foundational knowledge of their respective field (e.g. healthcare, social care, engineering, architecture, computer science, exercise physiology, etc.) and an intention to deepen their knowledge and skills in a more specialised area. Undergraduate students are also targeted, as knowledge areas relevant to HAA can be extrapolated from undergraduate related studies. Professionals in a variety of fields developing their skill and/or knowledge base in HAA through continuing education offerings are also of interest.

### Concept

This scoping review is designed to explore learning needs; defined as self-identified and personal specific interests or knowledge needs ^11^. In addition, all elements of the educational process are included as areas of interest, such as delivery, relevance, acceptability, and education outcome. These elements can offer insight into teacher-inferred learning needs, when self-identified student learning needs are not explicitly investigated. Together, data on learning needs, education relevance/acceptability, and educational outcomes can contribute to the formulation of appropriate learning outcomes.

### Context

Learning needs will be mapped in the context of HAA. As defined earlier, HAA considers healthy lifestyle promotion, consumption and nutrition practices as well as physical and social activity throughout the life course. An age-friendly society fosters HAA by building and maintaining intrinsic capacity across the life course and enabling greater functional ability in a person of any level of capacity.

## Methods

This scoping review was registered with the Open Science Framework ^12^. The conduct of this scoping review will follow the guidance published by the JBI ^10^.

### Search strategy

Electronic searches for relevant publications will be conducted in PubMed, EBSCO (Academic Search Complete), Scopus, and ASSIA, limited to publications after 1 ^st^ January 2000. Grey literature sources will initially include the first 50 results, ranked for relevance, of both Google Scholar and Research Gate searches. Publications deemed relevant from title and abstract screening will be eligible for full-text screening if published in English, Slovenian, Portuguese, Finnish, German, and Greek (as per the native fluency of the research team). Additional search methods will include forward and backward citation searching of included publications. The full search strategy is outlined in Table 2; terms within a box are combined using “OR” (e.g. “student OR professional”).

**Table 2.** Search strategy.

Details	Free text terms [title/abstract] and thesauri terms
1. Population: students	Student * ti,ab. Professional ti,ab.
2. Concept: learning needs	((learning or education * or training or knowledge) adj need *))ti,ab. ((learning or education * or training or profession * or knowledge) adj development))ti,ab. ((learning or education * or training or knowledge) adj relevance))ti,ab. ((learning or education * or training or knowledge) adj motivation))ti,ab. ((learning or education * or training or knowledge) adj competenc *))ti,ab. ((learning or education * or training or knowledge) adj requirement *))ti,ab. ((learning or education * or training or knowledge) adj delivery))ti,ab. ((learning or education * or training) adj process))ti,ab. (barrier * N5 facilitator *) N5 learning ti,ab. Education ti,ab. Competenc * ti,ab. CPD ti,ab. Continu * professional development ti,ab. Curricul * ti,ab.
3. Context: healthy and active ageing	((health * or success * or active or positive or productive or vital or resilient or robust or optimal or competent or effective or good or independent or authentic or strategic) adj aging ^~^))ti,ab. ((assist * living or assist * technolog *) AND aging ^~^) NOT machine learning))ti,ab. (aging ^~^ adj (society or well or productively or “in place”))ti,ab. Age friendly ti,ab. Aging ^~^ N5 wellbeing ti,ab.
Combination	1 AND 2 AND 3
Limit/filters	English, Slovenian, Portuguese, Finnish, German, Greek full-text language Published between 2000 and 1 July 2021

Key: *, truncation; adj, adjacent to; ~, alternative spellings; Nx, near within x words; ti,ab., title and abstract searches.

### Study selection

Records will be imported into Covidence, where duplicates not detected by the Covidence data management software will be manually removed by one reviewer. Remaining records will be screened by one reviewer, with independent screening being carried out by one of two other reviewers, first by title and abstract and subsequently by full-text. Any disagreements will be resolved through discussion, and, where necessary, a third reviewer.

### Extracting and charting results

Covidence will be used to manage citations and perform data extraction. A flow diagram using PRISMA-ScR guidelines ^13^ will be generated to report the selection process and all results. Data will be extracted for all included studies by one reviewer and checked by one of two other reviewers. Authors will be contacted for additional information, if required.

The following fields will be extracted from the included studies:

(1) Author(s)

(2) Year of publication

(3) Country of origin

(4) Area/field of study

(5) Study population

(5a) Students (UG, PG, CPD, other, N/A)

(5b) Stakeholders (learners, older persons, educators, N/A)

(6) Aims/purpose of study

(7) Methodology (e.g. type of study)

(8) Key findings

(8a) Type of programme/course

(8b) Content/curriculum

(8c) Learning needs identified, and process to identify

(8d) Learning delivery and assessment

(8e) Assessment of learning impact

(8f) Barriers/facilitators to learning

(8g) Evaluation of the programme (result and evaluator details)

During the screening process, it is possible that concept- and context-relevant publications with differing themes and publication types will arise, where these publications may not “fit” into the extraction criteria above. In this event, appropriate extraction criteria will be developed to present this information.

### Quality assessment

Methodological quality will be independently assessed by two reviewers in articles where a defined research process can be identified. A tool developed by Hawker and colleagues will be used as it allows for the critical appraisal of studies of quantitative, qualitative, and mixed-method designs ^14^. While quality assessment is not necessary for scoping reviews according to the JBI guidelines, it is recommended to improve the usefulness of the scoping review findings and to inform future research ^15^.

### Outcome presentation

As per the JBI methodology for scoping reviews ^10^, the results of this scoping review will be presented in diagrammatic or tabular form, supported by a description that is in line with the objective of the review. The results will be accompanied by a narrative summary.

### Study status

The current status of our study is that formal screening of search results against the eligibility criteria is ongoing.

## Data Availability

No data are associated with this article.
