# Supplementary material for: Postgraduate education in healthy and active ageing: learning needs, curriculum and expected outcomes: a scoping review protocol
Source: HRB Open Res. 2022 Oct 12;4:120. Originally published 2021 Nov 22. [Version 2] doi: 10.12688/hrbopenres.13444.2 (PMC10579848; doi:10.12688/hrbopenres.13444.2)
Supplement: Peer review history

# Reviewer report: Reviewer response for version 2

**DOI:** 10.21956/hrbopenres.14917.r33017

**Published:** 2023-10-17

**Authors:** Amy Jayne McKnight

**Affiliations:** 1 Queen's University, Belfast, UK

The authors have responded to my initial queries; however, their answers suggest that they have already completed their searches '.. *.had we found sources within our scope we would have broadened our search; this was not the case*'. Normally review protocols are published before data extraction starts to take account of external peer-review of the protocol. What is the current stage of this project?

 I would encourage this project to reconsider using JBI’s critical appraisal tools to assist in assessing the trustworthiness, relevance and results of published papers. There are a range of tools suited to individual types of publications that will be revealed from their search strategy. JBI's tools have been updated to reflect the content of different types of studies published more recently than the 2002 Hawker reference. Majid and colleague's 2018 review Appraising Qualitative Research for Evidence Syntheses may be of interest to this team ^1^.

Is the study design appropriate for the research question?

Partly

Is the rationale for, and objectives of, the study clearly described?

Yes

Are sufficient details of the methods provided to allow replication by others?

Yes

Are the datasets clearly presented in a useable and accessible format?

Not applicable

Reviewer Expertise:

molecular epidemiology, postgraduate research, medical & biomedical education in the UK, healthy ageing, rare disease

I confirm that I have read this submission and believe that I have an appropriate level of expertise to confirm that it is of an acceptable scientific standard.

## References

[1] : Appraising Qualitative Research for Evidence Syntheses: A Compendium of Quality Appraisal Tools. *Qual Health Res* .2018;28(13) : 10.1177/1049732318785358 2115-2131 10.1177/1049732318785358 30047306

# Reviewer report: Reviewer response for version 1

**DOI:** 10.21956/hrbopenres.14652.r31883

**Published:** 2022-05-03

**Authors:** Amy Jayne McKnight

**Affiliations:** 1 Queen's University, Belfast, UK

This scoping review protocol describes a proposed review to explore educational literature associated with health and active ageing. The topic is timely and of international interest given a worldwide focus promoting healthy ageing & the authors intention to develop an informed postgraduate degree in active ageing. The methods are acceptable – several queries are below to clarify the author’s plans and maximise transparency of the subsequent review.

 There have been more recent guidance published for scoping review protocols than the 2015 report cited here; I would encourage the authors to consider newer publications, ensure their scoping review complies with updated guidance and cite these in the protocol text.  E.g.

- Tricco, AC, Lillie, E, Zarin, W, O'Brien, KK, Colquhoun, H, Levac, D, Moher, D, Peters, MD, Horsley, T, Weeks, L, Hempel, S et al. PRISMA extension for scoping reviews (PRISMA-ScR): checklist and explanation. Ann Intern Med. 2018,169(7):467-473 ^1^.
-  Best practice guidance and reporting items for the development of scoping review  protocols. Peters MDJ, Godfrey C, McInerney P, Khalil H, Larsen P, Marnie C, Pollock D, Tricco AC, Munn Z.JBI Evid Synth. 2022 Apr 1;20(4):953-968. doi: 10.11124/JBIES-21-00242 ^2^

Similarly, the data appraisal method cited is from 2002 – how does that compare to more recently published methods such as those below?

- Institute JB. Critical appraisal tools 2020
- Tyndall J. AACODS Checklist - AACODS checklist (authority, accuracy, coverage, objectivity, date, significance for grey literature.

Is there a reason for Web of Science not being explicitly included in this review and limiting the grey literature search results to the first 50? Why are online grey literature databases such as GreyLit and OpenGrey not included in the search strategy? It is unusual to place such stringent limits on a broad scoping review that aims to, ‘ *establish the extent of research available’* as stated in the introduction. 

 Significant resources may have been published in the last two years – why does this 2022 protocol only consider publications from 2000-2020?

 It is surprising that this scoping review protocol has not been formally registered/submitted for registration to the Open Science Framework.  What are the dissemination plans for this review beyond informing the development of a new course?

Is the study design appropriate for the research question?

Partly

Is the rationale for, and objectives of, the study clearly described?

Yes

Are sufficient details of the methods provided to allow replication by others?

Yes

Are the datasets clearly presented in a useable and accessible format?

Not applicable

Reviewer Expertise:

molecular epidemiology, postgraduate research, medical & biomedical education in the UK, healthy ageing, rare disease

I confirm that I have read this submission and believe that I have an appropriate level of expertise to confirm that it is of an acceptable scientific standard, however I have significant reservations, as outlined above.

## References

[1] : Ethical, Legal and Social Issues of Digital Phenotyping as a Future Solution for Present-Day Challenges: A Scoping Review. *Sci Eng Ethics* .2021;28(1) : 10.1007/s11948-021-00354-1 1 10.1007/s11948-021-00354-1 34928438PMC8686352

[2] : Best practice guidance and reporting items for the development of scoping review protocols. *JBI Evid Synth* .2022;20(4) : 10.11124/JBIES-21-00242 953-968 10.11124/JBIES-21-00242 35102103

# Reviewer report: Reviewer response for version 1

**DOI:** 10.21956/hrbopenres.14652.r30880

**Published:** 2021-12-08

**Authors:** Janet Christine Frank

**Affiliations:** 1 UCLA Center for Health Policy Research, Fielding School of Public Health, University of California Los Angeles, Los Angeles, CA, USA

This submission focuses on the planned review protocol for a scoping review on learning needs, curriculum and expected outcomes for healthy and active aging and age friendly societies. This focus is important and will provide additional information for educators at the college and post-graduate levels. The purpose of a scoping review is well described. The planned methodology is sound and the planned data sources for extraction seem reasonable. The definitions for healthy and active aging, and age-friendly societies are appropriate. 

 It is well documented that content about healthy aging and age friendly societies is underutilized in all levels of education, and that much more needs to be included to address the current and future needs of the older adult population. Thus, it is important work to do and to report. As a Study Protocol article it is acceptable.

Is the study design appropriate for the research question?

Yes

Is the rationale for, and objectives of, the study clearly described?

Yes

Are sufficient details of the methods provided to allow replication by others?

Yes

Are the datasets clearly presented in a useable and accessible format?

No

Reviewer Expertise:

Competency-based geriatric and gerontology education; healthy aging curriculum development; educational program development and evaluation; diversity education; and behavioral health policy for older adults.

I confirm that I have read this submission and believe that I have an appropriate level of expertise to confirm that it is of an acceptable scientific standard.
